# BIOKID: Randomized controlled trial comparing bicarbonate and lactate buffer in biocompatible peritoneal dialysis solutions in children [ISRCTN81137991]

**DOI:** 10.1186/1471-2369-5-14

**Published:** 2004-10-14

**Authors:** Barbara Nau, Claus P Schmitt, Margarida Almeida, Klaus Arbeiter, Gianluigi Ardissino, Klaus E Bonzel, Alberto Edefonti, Michel Fischbach, Karin Haluany, Joachim Misselwitz, Markus J Kemper, Kai Rönnholm, Simone Wygoda, Franz Schaefer, European Pediatric Peritoneal Dialysis Study Group (EPPS)

**Affiliations:** 1 University Children's Hospital Heidelberg, Im Neuenheimer Feld 150, 69120 Heidelberg, Germany; 2 Hospital de Santa Maria, Serviço de Nefrologica Pediátrica, Av. Prof. Egas Moniz, 1649-035 Lisbon, Portugal; 3 Dept. of Pediatrics, AKH Wien, Währinger Gürtel 18–20, 1090 Vienna, Austria; 4 Division of Pediatric Nephrology, Istituti Clinici di Perfezionamento, Via della Commenda 9, 20122 Milan, Italy; 5 University Children's Hospital Essen, Hufelandstr. 55, 45122 Essen, Germany; 6 Nephrology Dialysis Transplantation Children's Unit, Hopitaux Universitaires de Strasbourg, Avenue Moliere, 67098 Strasbourg, France; 7 Urban Hospital St. Georg, Delitzscher Str. 141, 04129 Leipzig, Germany; 8 University Children's Hospital Jena, Kochstr. 2, 07740 Jena, Germany; 9 University Children's Hospital Hamburg, Martinistr. 52, 20246 Hamburg, Germany; 10 Hospital for Children and Adolescents, University of Helsinki, Stenbäckinkatu 11, 00290 Helsinki, Finland

## Abstract

**Background:**

Peritoneal dialysis (PD) is the preferred dialysis modality in children. Its major drawback is the limited technique survival due to infections and progressive ultrafiltration failure. Conventional PD solutions exert marked acute and chronic toxicity to local tissues. Prolonged exposure is associated with severe histopathological alterations including vasculopathy, neoangiogenesis, submesothelial fibrosis and a gradual loss of the mesothelial cell layer. Recently, more biocompatible PD solutions containing reduced amounts of toxic glucose degradation products (GDPs) and buffered at neutral pH have been introduced into clinical practice. These solutions contain lactate, bicarbonate or a combination of both as buffer substance. Increasing evidence from clinical trials in adults and children suggests that the new PD fluids may allow for better long-term preservation of peritoneal morphology and function. However, the relative importance of the buffer in neutral-pH, low-GDP fluids is still unclear. In vitro, lactate is cytotoxic and vasoactive at the concentrations used in PD fluids. The BIOKID trial is designed to clarify the clinical significance of the buffer choice in biocompatible PD fluids.

**Methods/design:**

The objective of the study is to test the hypothesis that bicarbonate based PD solutions may allow for a better preservation of peritoneal transport characteristics in children than solutions containing lactate buffer. Secondary objectives are to assess any impact of the buffer system on acid-base status, peritoneal tissue integrity and the incidence and severity of peritonitis.

After a run-in period of 2 months during which a targeted cohort of 60 patients is treated with a conventional, lactate buffered, acidic, GDP containing PD fluid, patients will be stratified according to residual renal function and type of phosphate binding medication and randomized to receive either the lactate-containing Balance solution or the bicarbonate-buffered Bicavera^® ^solution for a period of 10 months. Patients will be monitored by monthly physical and laboratory examinations. Peritoneal equilibration tests, 24-h dialysate and urine collections will be performed 4 times. Peritoneal biopsies will be obtained on occasion of intraabdominal surgery. Changes in small solute transport rates, markers of peritoneal tissue turnover in the effluent, acid-base status and peritonitis rates and severity will be analyzed.

## Background

Peritoneal dialysis (PD) is the preferred dialysis modality in children. Advantages of PD over hemodialysis relevant to pediatric patients include its compatibility with a normal lifestyle and full psychosocial integration, the continuous mode of blood purification without dysequilibrium conditions, the absence of vascular access issues and the avoidance of puncture pain. However, the major drawback of PD is its limited technique survival. Almost fifty percent of adult as well as pediatric PD patients must switch to hemodialysis within 4 to 5 years of treatment [1,2]. While the incidence of PD failure due to infectious complications is steadily decreasing, loss of ultrafiltration due to degenerative changes of the peritoneal tissue is becoming the leading cause of non-elective termination of PD [1]. Histopathological alterations induced by exposure to PD solutions include a severe vasculopathy, neoangiogenesis, submesothelial fibrosis and a progressive loss of the mesothelial cell layer [3-5]. Acute and chronic toxicity of standard PD fluids to mesothelial cells, affecting cell turnover and the pattern of growth factor and cytokine release, is considered a key mechanism underlying the progressive transformation of the peritoneum. Conventional PD fluids contain large doses of glucose, are lactate-buffered at acidic pH and contaminated with toxic glucose degradation products (GDP) formed during heat sterilization. Low pH, lactate and hyperosmolar glucose independently impair mesothelial cell functions [6-9]. GDPs impair the viability and functional integrity of mesothelial cells upon extended exposure [10], and stimulate VEGF and TGF-β release by mesothelial cells [11,12].

In recent years, a new generation of more biocompatible PD fluids has been introduced into clinical practice. The separation of alkaline and acidic fluid compartments in pluri-chamber bags permits to sterilize glucose at very low pH with greatly reduced GDP formation and yet produce pH-neutral final dialysis solutions, using lactate and/or bicarbonate as a buffer. We recently compared the safety and efficacy of Bicavera^® ^(Fresenius), a purely bicarbonate-buffered biocompatible PD solution, with that of a conventional acidic, lactate buffered solution by a three-month crossover trial in children on automated PD [13]. We observed a marked increase of the mesothelial cell marker CA125 in the effluent during Bicavera^® ^treatment, which was readily reversible when patients returned to conventional solution. This effect was also observed with lactate- or lactate/bicarbonate-buffered biocompatible PD solutions [14,15], and is interpreted as a functional and/or numeric recovery of mesothelial cells exposed to these fluids. Moreover, in agreement with previous studies [7,16] we observed a trend towards increasing small solute permeability with the standard solution; this trend was absent when Bicavera^® ^was used. Two studies observed a slightly lower initial increase of the functional peritoneal surface area during a single PD dwell with pH-neutral compared to acidic solutions compatible with reduced peritoneal capillary recruitment; this trend was significant in one study [17-19]. Fischbach et al. also demonstrated lower intraperitoneal pressure and less inflow pain in children receiving a low-GDP, neutral-pH PD solution [19]. Finally, we noted a more effective compensation of metabolic acidosis with Bicavera^® ^than with lactate buffered conventional fluid despite identical content of base equivalents.

While these results are encouraging with respect to the long term preservation of the peritoneal membrane and strongly favour the primary use of low-GDP, neutral-pH biocompatible PD fluids, the relative importance of the buffer system is still unclear. In vitro data suggest that lactate per se may compromise local cell functions independently of pH by affecting the cellular redox state and reducing cellular energy sources [6,20-22]. By intravital microcopy of rat peritoneum, lactate-based neutral-pH PD solution caused mesenteric vasodilation whereas bicarbonate buffered PD fluid had no hemodynamic effects [23]. All previous clinical trials comparing conventional and biocompatible PD fluids were unsuitable by design to identify any role of the buffer for peritoneal tissue integrity, perfusion and the acute or chronic regulation of peritoneal solute transport, since the solutions tested differed not only by the buffer used, but also by pH and GDP content. To clarify the role of the buffer the BIOKID trial has been designed. Patients participating in this trial will be exposed to two solutions which are both pH neutral and of low GDP content, but contain either pure bicarbonate or pure lactate as buffer compound.

## Methods/design

### Objectives of the study

The European Pediatric Peritoneal Dialysis Study Group (EPPS) plans a prospective, randomized study with administration of pH neutral, low-GDP PD solutions containing either lactate or bicarbonate buffer over a period of 10 months.

The primary objective is to evaluate the effect of lactate vs. bicarbonate buffer on peritoneal transport capacity in children. The hypothesis to be tested is that bicarbonate based PD solutions may allow for a significantly better preservation of peritoneal transport characteristics (D/P_Crea_) in children compared to a solution containing lactate buffer. Secondary objectives will be to assess differential effects of lactate and bicarbonate buffered PD fluids on acid-base status, surrogate parameters of peritoneal biocompatibility and local and systemic carbonyl stress, peritoneal morphology, the incidence and severity of peritonitis, statural growth and nutritional status. Moreover, this study will be used to assess genetic determinants of the peritoneal transporter status and the evolution of peritoneal morphology over time.

### Study design

This is a multicenter open-labelled, controlled, randomized clinical trial, designed to test the effects of the buffer substance in biocompatible PD fluids on peritoneal small solute transport capacity. All subjects will undergo a 2-month run-in period, in which they receive conventional lactate buffered, acidic, GDP containing PD fluid. During this period, patient eligibility for the trial will be verified and the dialysis dose will be optimized if necessary to ensure appropriate PD adequacy. At the end of this period, the patients will be stratified according to residual renal function (greater or less than 100 ml urine output/day/1.73 m^2^) and the type of phosphate binder therapy (Sevelamer vs. calcium-containing phosphate binders), since these variables may affect the overall efficacy of metabolic acidosis control. Following stratification, subjects will be randomized centrally to receive either the lactate-containing Balance solution or the bicarbonate buffered Bicavera^® ^solution for a period of 10 months. Both during the run-in period and during the intervention phase, patients will be monitored by monthly clinical and laboratory examinations, including capillary blood gas analyses. In addition, peritoneal equilibration tests (PETs), 24-hour dialysate and urine collections and intraabdominal pressure assessments will be performed at time of randomization (with conventional PD fluid) and after 3, 6 and 10 months of treatment (with the study solutions). Also, peritoneal biopsies will be performed on occasion of intraabdominal sugery or laparoscopy prior to start and after termination of the study (usually at time of catheter insertion and renal transplantation).

### Primary outcome measure

The primary outcome measure will be the longitudinal change in 4h-D/P_creatinine _in the sequential PET examinations. Differential changes in this parameter will indicate differences in the development of the peritoneal solute transport status over time.

### Secondary outcome measures

Secondary outcome measures will be surrogate parameters of mesothelial cell viability (CA-125), peritoneal neoangiogenesis (VEGF), fibrotic activity (TGF-β) and local inflammation (IL-6). With the same intention, the evolution of peritoneal histomorphology will be assessed in all patients available for sequential biopsies. Moreover, possible differential effects of lactate and bicarbonate buffer on the control of metabolic acidosis will be assessed by monthly blood gas analyses. Finally, the incidence and clinical course of peritonitis will be recorded as a possible indirect marker of local peritoneal macrophage function.

### Inclusion criteria

Criteria for inclusion in the study are 1) patients above 1 month and less than 19 years of age, 2) end-stage renal disease with manual or automated continuous peritoneal dialysis as maintenance treatment modality, 3) a fill volume approximately of 1100 ml/1.73 m^2 ^body surface area, 4) the most recent episode of PD-associated peritonitis, if any, occurred more than 3 weeks ago, 5) signed informed consent by parent/guardian, with a subject aged > 7 years also signing an age-appropriate assent form.

### Exclusion criteria

Criteria for exclusion from the study are 1) reduced efficiency of peritoneal dialysis due to anatomic anomalies or intraperitoneal adhesions, 2) uncontrolled hyperphosphatemia, 3) severe pulmonary, cardiac, hepatic or systemic disease including any kind of malignancy, and 4) current or recent (within 30 days) exposure to any investigational drug.

### Exit criteria

Reasons for permanently discontinuing the study medication are 1) renal transplantation, 2) switch to hemodialysis due to PD technique failure, 3) patient/parent withdrawal of consent of participate, 4) patient moving out of the area to a location with no participating center within reasonable distance, and 5) a severe adverse event.

### Study medications

The composition of the study fluids is given in Table 1. Both Bicavera^® ^and Balance will be available in three different glucose concentrations to meet individual ultrafiltration requirements. The fluids will be administered at a dose of approximately 1,100 ml/m^2 ^body surface area per dwell. Both in patients on CAPD and CCPD, the number of cycles and dwell times can be varied according to clinical needs. The dose of dialysis will be tailored individually in order to ascertain a minimum total weekly Kt/V_Urea _of ≥ 2.0.

**Table 1:** 

	Balance 1.5%	Balance 2.3%	Balance 4.25%	Bicavera^® ^1.5%	Bicavera^® ^2.3%	Bicavera^® ^4.25%
Sodium (mmol/l)	134	134	134	134	134	134
Calcium (mmol/l)	1.75	1.75	1.75	1.75	1.75	1.75
Magnesium (mmol/l)	0.5	0.5	0.5	0.5	0.5	0.5
Chloride (mmol/l)	101.5	101.5	101.5	104.5	104.5	104.5
L-Lactate (mmol/l)	35	35	35	-	-	-
Bicarbonate (mmol/l)	-	-	-	34	34	34
Glucose-monohydrate (g/l)	15	23	42.5	15	23	42.5
Osmolarity (mosmol/l)	358	401	511	358	399	509
PH	7.4	7.4	7.4	7.4	7.4	7.4

Any kind of concomitant medication during the run-in period and the study period will be documented in the case report form with respect to type, dosage and mode of delivery. In case of peritonitis (defined by cloudy effluent, white blood cell count greater than 100/mm^3 ^with more than 50% polymorphonuclear leukocytes), treatment will be given intraperitoneally according to international pediatric guidelines [24] using cefazoline and ceftazidime in patients with mild peritonitis and a glycopeptide/ceftazidime combination in patients with defined risk factors for severe course and poor outcome.

Bicarbonate supplementation will be discontinued at start of the run-in period and only re-instituted if blood bicarbonate levels drop below 17 mmol/l despite sufficient dialysis efficacy. The recommended dosage is 0.5 mmol/kg/day divided into 3 doses.

### Clinical safety monitoring

An adverse event is defined as any untoward medical occurence in a patient who takes the study medication. It does not necessarily have a causal relationship with this treatment. This may be an unfavourable and unintended sign, symptom or disease, which is observed after exposure to the study medication, whether or not considered related to the treatment. Moreover, the participating investigators will report all treatment-emergent adverse events that are observed on the online adverse event form. This applies regardless of the clinical significance or the assessment of study drug causality. In this trial, such adverse events may include inflow pain, severe changes of the state of hydration, abnormal electrolyte and glucose blood levels, peritonitis, abdominal hernia, and allergic reactions.

A severe adverse event or reaction is any untoward medical occurrence that a) results in death, b) is life-threatening, c) requires inpatient hospitalization or prolonges an existing hospitalization, or d) results in persistent or significant disability/incapacity.

Any serious adverse event, whether or not considered related to the study medication, and any unexpected drug reactions with significant hazard to the patient population will be reported to the responsible safety assessor at Fresenius Medical Care by phone or by fax within 24 hours following first knowledge of the event. Alternatively the clinical monitor may be informed. This information will be forwarded to and evaluated by the safety monitoring committee, and reports of serious adverse reactions will be disseminated to all participating centers for submission to their respective institutional review boards. Fresenius Medical Care is responsible for passing on the information to relevant supervisory authorities.

### Data management

Data acquisition will be entirely through the internet. The case report form menus will be used to record the following: 1) baseline clinical patient information, 2) physical and biochemical examination variables, 3) study and concurrent medications, and 4) adverse events.

Periodic computerized audit reports will be run to monitor data quality and completeness. The data base is stored on a server drive that is backed up to tape daily by Tel-A-Vision, Media Networking GmbH.

In order to insure confidentiality, data from each patient will be recorded in the computer data base with a unique contributing center code, study code, sequence number and patient initials. Patient names are never entered online or forwarded in any other form to the coordinating office.

### Sample size estimation

The primary study outcome is the change in 4h-D/P_Cr _from the time of randomization to the conclusion of the 10-month study period. In a previous trial we demonstrated a 6% increase of D/P_Cr _in children on lactate-buffered PD fluid in contrast to a 4% decrease in D/P_Cr _using bicarbonate buffered fluid within 3 months of exposure, resulting in a statistically significant 10% difference in the evolution of peritoneal creatinine transport rate [13]. Assuming that this effect was at least in part due to the different buffer substances applied, a difference in D/P_Cr _at least 7 ± 10% can be expected when Balance and Bicavera^® ^are applied for 10 months. To detect this difference with a sensitivity of 80% and an error probability of 5%, at least 15 patients per randomization group will be required. Assuming a 50% drop-out rate due to renal transplantation and other reasons in the course of the study, 60 patients will have to be enrolled.

### Statistical approach

A computer based randomization protocol will be generated and applied centrally at the end of the run-in period. Baseline comparability between the two treatment groups will be evaluated with respect to entry criteria. Chi-square and t-tests will be used to assess differences between the two groups on the baseline variables. Any variables that are found to be discrepant between the two groups and that are related to the outcome variables will be treated as co-variates in later analyses.

In order to evaluate the patients' change in D/P_Cr _ratios, two strategies will be employed:

1. Repeated measure ANOVA will be performed on those patients who complete the 10-month observation period on the study medications.

2. The Kaplan-Meier method will be used to estimate the time in which patients are likely to display a ≥ 7.5 % increase in the D/P_Crea _ratio during the 10-month period. All patients started on study medication will be available for this analytical approach.

The same strategies will be used to analyze secondary outcome variables.

### Publication of study results

All publications will be authored by members of the scientific advisory committee. Co-authors will have contributed to the design, analysis, execution and actual reporting of the study.

### Study investigators

Steering committee: C.P. Schmitt, F. Schaefer, K.E. Bonzel, K. Rönnholm

Scientific advisory committee: M. Almeida, K. Arbeiter, G. Ardissino, K.E. Bonzel, A. Edefonti, M. Fischbach, K. Haluany, J. Misselwitz, Markus J. Kemper, K. Rönnholm, F. Schaefer, C.P. Schmitt, S. Wygoda

Safety monitoring committee: V. Schwenger, U. Querfeld, G. Offner

## Discussion

We report the protocol of a randomized clinical trial designed to test the effect of the buffer type on the evolution of peritoneal tissue integrity and transport function in children treated with 'biocompatible', i.e. neutral-pH, low-GDP PD solutions. The study utilizes the availability of two novel biocompatible PD solutions manufactured by the same company which differ selectively in the buffer employed, namely either pure lactate or pure bicarbonate. The comparative administration of these solutions will provide information on differences in peritoneal cell viability, tissue morphology, local host defense and solute transfer capacity potentially inferred by cytotoxic and/or vasocative effects of lactate administered to the abdominal cavity in unphysiological concentrations.

## Competing interests

This investigator-initiated trial was designed exclusively by the members of the EPPS scientific advisory committee. None of the investigators have any financial relationship with the manufacturer of the study medication. Financial support is received from Fresenius Medical Care to cover coordination costs and investigator meetings.

## Pre-publication history

The pre-publication history for this paper can be accessed here:



## References

[B1] Davies SJ, Phillips L, Griffiths SM, Russell LH, Naish PF, Russell GI (1998). What really happens to people on long-term peritoneal dialysis?. Kidney International.

[B2] Schaefer F, Klaus G, Müller-Wiefel DE, Mehls O, (MEPPS) Mid European Pediatric Peritoneal Dialysis Study Group (1999). Current practice of peritoneal dialysis in children: results of a longitudinal survey. Perit Dial Int.

[B3] Williams JD, Craig KJ, Topley N, Von Ruhland C, Fallon M, Newman GR, Mackenzie RK, Williams GT (2002). Morphologic changes in the peritoneal membrane of patients with renal disease. J Am Soc Nephrol.

[B4] Schneble F, Bonzel KE, Waldherr R, Bachmann S, Roth H, Scharer K (1992). Peritoneal morphology in children treated by continuous ambulatory peritoneal dialysis.. Pediatr Nephrol.

[B5] Dobbie JW, Anderson JD, Hind C (1994). Long term effects of peritoneal dialysis on peritoneal morphology. Perit Dial Int.

[B6] Witowski J, Topley N, Jorres A, Liberek T, Coles GA, Williams JD (1995). Effect of lactate-buffered peritoneal dialysis fluids on human peritoneal mesothelial cell interleukin-6 and prostaglandin synthesis. Kidney Int.

[B7] Davies SJ, Phillips L, Naish PF, Russell GI (2001). Peritoneal glucose exposure and changes in membrane solute transport with time on peritoneal dialysis. J Am Soc Nephrol.

[B8] Krediet RT, Lindholm B, Rippe B (2000). Pathophysiology of peritoneal membrane failure. Perit Dial Int.

[B9] Yanez-Mo M, Lara-Pezzi E, Selgas R, Ramirez-Huesca M, Dominguez-Jimenez C, Jimenez-Heffernan JA, Aguilera A, Sanchez-Tomero JA, Bajo MA, Alvarez V, Castro MA, del Peso G, Cirujeda A, Gamallo C, Sanchez-Madrid F, Lopez-Cabrera M (2003). Peritoneal dialysis and epithelial-to-mesenchymal transition of mesothelial cells. N Engl J Med.

[B10] Witowski J, Wisniewska J, Korybalska K, Bender TO, Breborowicz A, Gahl GM, Frei U, Passlick-Deetjen J, Jorres A (2001). Prolonged exposure to glucose degradation products impairs viability and function of human peritoneal mesothelial cells. J Am Soc Nephrol.

[B11] Inagi R, Miyata T, Yamamoto T, Suzuki D, Urakami K, Saito A, van Ypersele de Strihou C, Kurokawa K (1999). Glucose degradation product methylglyoxal enhances the production of vascular endothelial growth factor in peritoneal cells: role in the functional and morphological alterations of peritoneal membranes in peritoneal dialysis. FEBS Lett.

[B12] Kang DH, Hong YS, Lim HJ, Choi JH, Han DS, Yoon KI (1999). High glucose solution and spent dialysate stimulate the synthesis of transforming growth factor-beta1 of human peritoneal mesothelial cells: effect of cytokine costimulation.. Perit Dial Int.

[B13] Haas S, Schmitt CP, Bonzel KE, Pieper AK, Fischbach M, John U, Arbeiter K, Schaup TP, Passlick-Deetjen J, Mehls O, Schaefer F (2003). Improved acidosis correction and recovery of mesothelial cell mass with neutral-pH bicarbonate dialysis solution among children undergoing automated peritoneal dialysis.. J Am Soc Nephrol.

[B14] Jones S, Holmes CJ, Krediet RT, Mackenzie R, Faict D, Tranaeus A, Williams JD, Coles GA, Topley N (2001). Bicarbonate/lactate-based peritoneal dialysis solution increases cancer antigen 125 and decreases hyaluronic acid levels. Kidney Int.

[B15] Rippe B, Simonsen O, Heimbürger O, Christensson A, Haraldsson B, Stelin G, Weiss J, Nielsen FD, Bro S, Friedberg M, Wieslander A (2001). Long-term clinical effects of a peritoneal dialysis fluid with less glucose degradation products. Kidney Int.

[B16] Wang T, Cheng HH, Liu SM, Wang Y, Wu JL, Peng WX, Zhong JH, Lindholm B (2001). Increased peritoneal membrane permeability is associated with abnormal peritoneal surface layer.. Perit Dial Int.

[B17] Schmitt CP, Haraldsson B, Doetschmann R, Zimmering M, Greiner C, Böswald M, Klaus G, Passlick-Deetjen J, Schaefer F (2002). Effects of pH-neutral, bicarbonate-buffered dialysis fluid on peritoneal transport kinetics in children. Kidney Int.

[B18] Fischbach M, Terzic J, Chauvé S, Laugel V, Muller A, Haraldsson B (2004). Effect of peritoneal dialysis fluid composition on peritoneal area available for exchange in children. Nephrol Dial Transplant.

[B19] Fischbach M, Haraldsson B, Helms P, Danner S, Laugel V, Terzic J (2003). The peritoneal membrane: a dynamic dialysis membrane in children. Adv Perit Dial.

[B20] Breborowicz A, Rodela H, Martis L, Oreopoulos DG (1996). Intracellular glutathione in human peritoneal mesothelial cells exposed in vitro to dialysis fluid.. Int J Artif Organs.

[B21] Liberek T, Topley N, Jorres A, Petersen MM, Coles GA, Gahl GM, Williams JD (1993). Peritoneal dialysis fluid inhibition of polymorphonuclear leukocyte respiratory burst activation is related to the lowering of intracellular pH. Nephron.

[B22] Plum J, Rezeghi P, Lordnejad RM, Perniok A, Fleisch M, Fussholler A, Schneider M, Grabensee B (2001). Peritoneal dialysis fluids with a physiologic pH based on either lactate or bicarbonate buffer-effects on human mesothelial cells. Am J Kidney Dis.

[B23] Mortier S, De Vriese AS, Van de Voorde J, Schaub TP, Passlick-Deetjen J, Lameire NH (2002). Hemodynamic effects of peritoneal dialysis solutions on the rat peritoneal membrane: role of acidity, buffer choice, glucose concentration, and glucose degradation products. J Am Soc Nephrol.

[B24] Warady BA, Schaefer F, Holloway M, Alexander S, Kandert M, Piraino B, Salusky I, Tranaeus A, Divino J, Honda M, Mujais S, Verrina E (2000). Consensus guidelines for the treatment of peritonitis in pediatric patients receiving peritoneal dialysis.. Perit Dial Int.

